# Supplementary material for: Implementing postpartum family planning services in rural Rwanda: A mixed-methods study
Source: PLoS One. 2025 Dec 30;20(12):e0338627. doi: 10.1371/journal.pone.0338627 (PMC12752963; doi:10.1371/journal.pone.0338627)
Supplement: S1 Fig — (DOCX) [file pone.0338627.s001.docx]

**Supplement 1. Code tree guiding focus group themes and sub-themes**

**Access to PPFP counseling**

**Strategies to engage male partners in PPFP**

**Concerns about PPFP**

**Knowledge about PPFP benefits**

**PPFP knowledge limitations**

- Couple educational counseling
- Educational counseling in the community or at home
- Encourage male partners to learn about PPFP methods, benefits, and support their wives during decision making
- Concerns about FP method side effects
- Concerns about FP method failure
- Concerns about FP method contraindications
- Lack of male partner involvement, concerns about men’s
- Religious concerns and societal opinions against FP
- Financial concerns
  - Cost of removal
  - Cost of side effects
  - Lack of health insurance
- Lack of information on how FP methods work
- Lack of IUD/PPIUD awareness
- Birth spacing for family well-being
- Better child growth
- Beneficial for family finances
- Prevent ion of unintended pregnancy
- Educational counseling at the health center (during ANC, at delivery, infant vaccination)
- Education counseling delivered by a community health worker

FP: family planning; PPFP: postpartum family planning; ANC: antenatal care; IUD: intrauterine device; PPIUD: postpartum intrauterine device
