# Supplementary material for: Implementing postpartum family planning services in rural Rwanda: A mixed-methods study
Source: PLoS One. 2025 Dec 30;20(12):e0338627. doi: 10.1371/journal.pone.0338627 (PMC12752963; doi:10.1371/journal.pone.0338627)
Supplement: S1 Appendix — (DOCX) [file pone.0338627.s002.docx]

**Appendix 1: COREQ (COnsolidated criteria for REporting Qualitative research) Checklist.**

A checklist of items that should be included in reports of qualitative research. You must report the page number in your manuscript where you consider each of the items listed in this checklist. If you have not included this information, either revise your manuscript accordingly before submitting or note N/A.

| **Topic** | **Item no.** | **Guide questions/description** | **Reported on page no.** |
| --- | --- | --- | --- |
| **Domain 1: research team and reflexivity** | | |  |
| *Personal characteristics* | | |  |
| Interviewer/facilitator | 1 | Which author/s conducted the interview or focus group?  ***Research nurses from Center for Family Health Research (CFHR) trained*** | 10 |
| Credentials | 2 | What were the researcher’s credentials? e.g., PhD, MD  ***CFHR personnel – MD/MPH***  ***CFHR research nurses –Registered Nurses (RN) with nursing diploma and additional qualification of Bachelor of Social Science or Public health*** | 10 |
| Occupation | 3 | What was their occupation at the time of the study?  ***CFHR/Projet San Francisco (PSF) personnel/staff*** | 1 |
| Gender | 4 | Was the researcher male or female?  ***Female*** | 1 |
| Experience and training | 5 | What experience or training did the researcher have?  ***CFHR research nurses were trained about the study and experienced in conducting FGDs.*** | 10 |
| *Relationship with participants* | | |  |
| Relationship established | 6 | Was a relationship established prior to study commencement?  ***CFHR staff have worked previously in the selected health facilities, specifically recruiting study participants from ANC for an Ebola safety vaccine trial.*** | 8 |
| Participant knowledge of the interviewer | 7 | What did the participants know about the researcher? e.g., personal goals, reasons for doing the research.  ***The purpose and procedures of the study was explained to the participants before the start of the focus group discussion*** | 10 |
| Interviewer characteristics | 8 | What characteristics were reported about the interviewer/facilitator? e.g., bias, assumptions, reasons and interests in the research topic  ***Only gender and professional credentials are reported. We note in the limitations section that we have not collected data on biases or assumptions held by the CFHR interviewers.*** | 10 |
| **Domain 2: study design** | | |  |
| *Theoretical framework* | | |  |
| Methodological orientation and theory | 9 | What methodological orientation was stated to underpin the study? e.g., grounded theory, discourse analysis, ethnography, phenomenology, content analysis  ***Our focus group discussion (FGD) guides are based on a qualitative content analysis with a deductive approach.*** | 10 |
| *Participant selection* | | |  |
| Sampling | 10 | How were participants selected? e.g., purposive, convenience  ***Convenience*** | 8 |
| Method of approach | 11 | How were participants approached? e.g., face-to-face, telephone, mail, email  ***The participants were approached face-to-face during their routine antenatal care service.*** | 8 |
| Sample size | 12 | How many participants were in the study?  ***27 couples were included in the FGDs.*** | 17 |
| Non-participation | 13 | How many people refused to participate or dropped out? Reasons?  ***All participants who were recruited for the focus group discussion provided informed consent and participated.*** | 18 |

**Appendix 1 (continued):** COREQ (COnsolidated criteria for REporting Qualitative research) Checklist.

| **Topic** | **Item no.** | **Guide questions/description** | **Reported on page no.** |
| --- | --- | --- | --- |
| **Domain 2: study design** | |  |  |
| *Setting* | |  |  |
| Setting of data collection | 14 | Where was the data collected? e.g., home, clinic, workplace  ***Health centers*** | 9 |
| Presence of non-participants | 15 | Was anyone else present besides the participants and researchers?  **No** |  |
| Description of sample | 16 | What are the important characteristics of the sample? e.g., demographic data, date  ***The demographic data* are reported in Table 3.** | 19 |
| *Data collection* | |  |  |
| Interview guide | 17 | Were questions, prompts, guides provided by the authors? Was it pilot tested?  ***The focus group guide was adapted based on guides developed for use in prior studies using the health belief model.*** | 10 |
| Repeat interviews | 18 | Were repeat interviews carried out? If yes, how many?  ***No repeat interviews were conducted.*** |  |
| Audio/visual recording | 19 | Did the research use audio or visual recording to collect the data?  ***The focus group discussions were audio-recorded.*** | 10 |
| Field notes | 20 | Were field notes made during and/or after the interview or focus group?  ***Field notes were made during the group discussion.*** | 10 |
| Duration | 21 | What was the duration of the interviews or focus group?  ***The duration was ranging between 1 hour 45 minutes to 2 hours 15 minutes*** | 10 |
| Data saturation | 22 | Was data saturation discussed?  ***Yes*** | 27 |
| Transcripts returned | 23 | Were transcripts returned to participants for comment and/or correction?  ***No*** | 11 |
| **Domain 3: analysis and findings** | |  |  |
| *Data analysis* | |  |  |
| Number of data coders | 24 | How many data coders coded the data?  ***None; no coding was conducted.*** | 10-11 |
| Description of the coding tree | 25 | Did authors provide a description of the coding tree?  ***Not applicable*** |  |
| Derivation of themes | 26 | Were themes identified in advance or derived from the data?  ***Themes were identified in advance.*** | 10-11 |
| Software | 27 | What software, if applicable, was used to manage the data?  ***NVivo*** | 10 |
| Participant checking | 28 | Did participants provide feedback on the findings?  ***No*** | 11 |
| *Reporting* | |  |  |
| Quotations presented | 29 | Were participant quotations presented to illustrate the themes/findings? Was each quotation identified? e.g., participant number  ***Quotes are presented to illustrate the themes; quotes are not identified by participant number because participant number was not linked to any demographics; this is now noted in the limitations section.*** | 20-24 |

**Appendix 1 (continued):** COREQ (COnsolidated criteria for REporting Qualitative research) Checklist.

| **Topic** | **Item no.** | **Guide questions/description** | **Reported on page no.** |
| --- | --- | --- | --- |
| **Domain 3: analysis and findings** | |  |  |
| *Reporting* | |  |  |
| Data and findings consistent | 30 | Was there consistency between the data presented and the findings?  ***Yes*** | 12-24 |
| Clarity of major themes | 31 | Were major themes clearly presented in the findings?  ***Yes*** | 20-24 |
| Clarity of minor themes | 32 | Is there a description of diverse cases or discussion of minor themes?  ***Yes*** | 20-24 |

Developed from: Tong A, Sainsbury P, Craig J. Consolidated criteria for reporting qualitative research (COREQ): a 32-item checklist for interviews and focus groups. Int J Qual Health Care. 2007 Dec;19(6):349357. doi: 10.1093/intqhc/mzm042.

**Once you have completed this checklist, please save a copy and upload it as part of your submission. DO NOT include this checklist as part of the main manuscript document. It must be uploaded as a separate file**
