# Supplementary material for: Implementing postpartum family planning services in rural Rwanda: A mixed-methods study
Source: PLoS One. 2025 Dec 30;20(12):e0338627. doi: 10.1371/journal.pone.0338627 (PMC12752963; doi:10.1371/journal.pone.0338627)
Supplement: Attachment — Submitted filename: 0.PONE-D-24-28650.pdf [file pone.0338627.s003.pdf]

PLOS ONE

Implementing postpartum family planning services in rural Rwanda: a mixed-methods study

--Manuscript Draft--

Manuscript Number:	PONE-D-24-28650
Article Type:	Research Article
Full Title:	Implementing postpartum family planning services in rural Rwanda: a mixed-methods study
Short Title:	Rural postpartum family planning in Rwanda
Corresponding Author:	Kristin M. Wall, Ph.D.<br>Emory University School of Public Health<br>Atlanta, UNITED STATES OF AMERICA
Keywords:	Postpartum; contraception; family planning; couples; assessment of health care needs; Rwanda; intrauterine device
Abstract:	Introduction: Postpartum family planning (PPFP) is key to reducing significant maternal-child adverse outcomes related to short interpregnancy intervals and unintended pregnancies. This mixed-method study evaluated PPFP needs in rural government health facilities as well as client knowledge and barriers to PPFP uptake in Rwanda.<br>Methods: From May-July 2023, we conducted cross-sectional PPFP needs assessments in twelve rural government health facilities (two hospitals, four health centers, six health posts) and focus group discussions (FGDs) among couples attending antenatal clinics to understand women's and men's perceptions and barriers to PPFP uptake.<br>Results : Seventeen (65%) hospital nurses and 11 (23%) health center nurses were trained in implant insertion, and 6 (23%) hospital nurses and 4 (9%) health center nurses were trained in postpartum IUD (PPIUD) insertion. Hospitals provided an average of 204 postpartum implants (29% per delivery) and 7 PPIUDs per month(1% per delivery), while health centers provided 25 postpartum implants and no PPIUDs per month. At health posts, there was no equipment for implant or IUD provision. The majority of the 27 couples recruited for FGDs (93% of women, 74% of men) had received family planning educational counseling though less than half (41%) of couples reported learning about the PPIUD. Participants reported concerns about the lack of information on how family planning works and side effects.<br>Conclusions: Our study highlights the need to enhance PPFP training and provision in rural areas, especially for PPIUD, and confirmed a low postpartum LARC uptake. Knowledge gaps and concerns about side effects were emphasized in FGDs. PPFP demand creation strategies tailored for the rural populace as well as provider training could improve PPFP uptake in rural government clinics of Rwanda. Rural health posts could be explored as potential locations for PPFP counseling.
Order of Authors:	Claudine Umuhoza
	Mame Diarra Niang
	Rosine Ingabire
	Julien Nyombayire
	Amelia Mazzei
	Rachel Parker
	Amanda Tichacek
	Azhar Nizam
	Jessica Sales
	Lisa Haddad
	Phaedra Corso

Powered by Editorial Manager® and ProduXion Manager® from Aries Systems Corporation

	Susan Allen
	Kristin M. Wall, Ph.D.
	Etienne Karita
**Additional Information:**	
**Question**	**Response**
**Financial Disclosure**<br><br>Enter a financial disclosure statement that describes the sources of funding for the work included in this submission. Review the submission guidelines for detailed requirements. View published research articles from *PLOS ONE* for specific examples.<br><br>This statement is required for submission and **will appear in the published article** if the submission is accepted. Please make sure it is accurate.<br><br>**Funded studies**<br>Enter a statement with the following details:<br>• Initials of the authors who received each award<br>• Grant numbers awarded to each author<br>• The full name of each funder<br>• URL of each funder website<br>• Did the sponsors or funders play any role in the study design, data collection and analysis, decision to publish, or preparation of the manuscript?<br><br>Did you receive funding for this work?	Yes
Please add funding details.<br>as follow-up to "**Financial Disclosure**<br><br>Enter a financial disclosure statement that describes the sources of funding for the work included in this submission. Review the submission guidelines for detailed requirements. View published research articles from *PLOS ONE* for specific examples.<br><br>This statement is required for submission and **will appear in the published article** if	National Institutes of Health (https://nih.gov/) R01HD101600 to KMW. Support was also available from the Emory University Global Field Experience program (https://sph.emory.edu/rollins-life/community-engaged-learning/global-field-experience/index.html) to MDN. The funders had no role in the study design; collection, management, analysis, or interpretation of data; writing of the report; or the decision to submit the report for publication.

the submission is accepted. Please make sure it is accurate.<br><br>**Funded studies**<br>Enter a statement with the following details:<br>• Initials of the authors who received each award<br>• Grant numbers awarded to each author<br>• The full name of each funder<br>• URL of each funder website<br>• Did the sponsors or funders play any role in the study design, data collection and analysis, decision to publish, or preparation of the manuscript?<br><br>Did you receive funding for this work?"	
Please select the country of your main research funder (please select carefully as in some cases this is used in fee calculation).<br>as follow-up to "**Financial Disclosure**<br><br>Enter a financial disclosure statement that describes the sources of funding for the work included in this submission. Review the submission guidelines for detailed requirements. View published research articles from *PLOS ONE* for specific examples.<br><br>This statement is required for submission and **will appear in the published article** if the submission is accepted. Please make sure it is accurate.<br><br>**Funded studies**<br>Enter a statement with the following details:<br>• Initials of the authors who received each award<br>• Grant numbers awarded to each author<br>• The full name of each funder<br>• URL of each funder website<br>• Did the sponsors or funders play any role in the study design, data collection and analysis, decision to publish, or preparation of the manuscript?	UNITED STATES - US

Did you receive funding for this work?"	
**Competing Interests**<br><br>Use the instructions below to enter a competing interest statement for this submission. On behalf of all authors, disclose any competing interests that could be perceived to bias this work—acknowledging all financial support and any other relevant financial or non-financial competing interests.<br><br>This statement is required for submission and **will appear in the published article** if the submission is accepted. Please make sure it is accurate and that any funding sources listed in your Funding Information later in the submission form are also declared in your Financial Disclosure statement.<br><br>View published research articles from *PLOS ONE* for specific examples.<br><br>**NO authors have competing interests**<br><br>Enter: *The authors have declared that no competing interests exist.*<br><br>**Authors with competing interests**<br><br>Enter competing interest details beginning with this statement:<br><br>*I have read the journal's policy and the authors of this manuscript have the following competing interests: [insert competing interests here]*<br><br>* typeset	The authors have declared that no competing interests exist.
**Ethics Statement**<br><br>Enter an ethics statement for this submission. This statement is required if the study involved:<br><br>• Human participants<br>• Human specimens or tissue	Ethical approval to conduct focus group discussions (FGDs) was obtained from the Rwanda National Ethics Committee and Emory IRB, and written informed consent from eligible couples was obtained before allowing them to participate in any FGD and each participant was compensated for the time.

• Vertebrate animals or cephalopods<br>• Vertebrate embryos or tissues<br>• Field research<br><br>Write "N/A" if the submission does not require an ethics statement.<br><br>General guidance is provided below. Consult the submission guidelines for detailed instructions. **Make sure that all information entered here is included in the Methods section of the manuscript.**	

Powered by Editorial Manager® and ProduXion Manager® from Aries Systems Corporation

**Format for specific study types**<br><br>**Human Subject Research (involving human participants and/or tissue)**<br>• Give the name of the institutional review board or ethics committee that approved the study<br>• Include the approval number and/or a statement indicating approval of this research<br>• Indicate the form of consent obtained (written/oral) or the reason that consent was not obtained (e.g. the data were analyzed anonymously)<br><br>**Animal Research (involving vertebrate animals, embryos or tissues)**<br>• Provide the name of the Institutional Animal Care and Use Committee (IACUC) or other relevant ethics board that reviewed the study protocol, and indicate whether they approved this research or granted a formal waiver of ethical approval<br>• Include an approval number if one was obtained<br>• If the study involved *non-human primates*, add *additional details* about animal welfare and steps taken to ameliorate suffering<br>• If anesthesia, euthanasia, or any kind of animal sacrifice is part of the study, include briefly which substances and/or methods were applied<br><br>**Field Research**<br><br>Include the following details if this study involves the collection of plant, animal, or other materials from a natural setting:<br>• Field permit number<br>• Name of the institution or relevant body that granted permission	
**Data Availability**<br><br>Authors are required to make all data underlying the findings described fully available, without restriction, and from the time of publication. PLOS allows rare exceptions to address legal and ethical concerns. See the PLOS Data Policy and FAQ for detailed information.	Yes - all data are fully available without restriction

A Data Availability Statement describing where the data can be found is required at submission. Your answers to this question constitute the Data Availability Statement and **will be published in the article**, if accepted.<br><br>**Important:** Stating 'data available on request from the author' is not sufficient. If your data are only available upon request, select 'No' for the first question and explain your exceptional situation in the text box.<br><br>Do the authors confirm that all data underlying the findings described in their manuscript are fully available without restriction?	
**Describe where the data may be found in full sentences. If you are copying our sample text, replace any instances of XXX with the appropriate details.**<br><br>• If the data are **held or will be held in a public repository**, include URLs, accession numbers or DOIs. If this information will only be available after acceptance, indicate this by ticking the box below. For example: *All XXX files are available from the XXX database (accession number(s) XXX, XXX.)*.<br>• If the data are all contained **within the manuscript and/or Supporting Information files**, enter the following: *All relevant data are within the manuscript and its Supporting Information files.*<br>• If neither of these applies but you are able to provide **details of access elsewhere**, with or without limitations, please do so. For example:<br><br>*Data cannot be shared publicly because of [XXX]. Data are available from the XXX Institutional Data Access / Ethics Committee (contact via XXX) for researchers who meet the criteria for access to confidential data.*<br><br>*The data underlying the results presented in the study are available from (include the name of the third party*	The data will be available at Harvard Dataverse (https://doi.org/10.7910/DVN/OWFLDN) upon submission.

and contact information or URL).<br>• This text is appropriate if the data are owned by a third party and authors do not have permission to share the data.<br><br>* typeset	
Additional data availability information:	Tick here if the URLs/accession numbers/DOIs will be available only after acceptance of the manuscript for publication so that we can ensure their inclusion before publication.

Powered by Editorial Manager® and ProduXion Manager® from Aries Systems Corporation

Manuscript
Click here to access/download;Manuscript;PPFPRural_11Jul24_ForSubmissi

Implementing postpartum family planning services in rural Rwanda: a mixed-methods study

Short title: Rural postpartum family planning in Rwanda

Claudine Umuhoza, Projet San Francisco (PSF)/Center for Family Health Research (CFHR), Kigali, Rwanda

Mame Diarra Niang, Department of Epidemiology, School of Public Health, Emory University

Rosine Ingabire, Projet San Francisco (PSF)/Center for Family Health Research (CFHR), Kigali, Rwanda

Julien Nyombayire, Projet San Francisco (PSF)/Center for Family Health Research (CFHR), Kigali, Rwanda

Amelia Mazzei, Department of Pathology, School of Medicine, Emory University; PSF/CFHR, Kigali, Rwanda

Rachel Parker, Department of Pathology, School of Medicine, Emory University

Amanda Tichacek, Department of Pathology, School of Medicine, Emory University

Azhar Nizam, Department of Biostatistics and Bioinformatics, School of Public Health, Emory University

Jessica M. Sales, Department of Behavioral, Social and Health Education Sciences, School of Public Health, Emory University

Lisa B. Haddad, Population Council, Center for Biomedical Research, New York, NY

Phaedra Corso, Office of Research, Department of Health Policy, Kennesaw State University

Susan Allen, Department of Pathology, School of Medicine, Emory University

Etienne Karita, Department of Pathology, School of Medicine, Emory University; PSF/CFHR, Kigali, Rwanda

Kristin M. Wall, Department of Epidemiology, School of Public Health, Emory University

Funding: National Institutes of Health, NICHD, R01HD101600. The National Institutes of Health had no role in the study design; collection, management, analysis, or interpretation of data; writing of the report; or the decision to submit the report for publication. Support was also available from the Emory University Global Field Experience program.

Abstract

Introduction: Postpartum family planning (PPFP) is key to reducing significant maternal-child adverse outcomes related to short interpregnancy intervals and unintended pregnancies. This mixed-method study evaluated PPFP needs in rural government health facilities as well as client knowledge and barriers to PPFP uptake in Rwanda.

Methods: From May-July 2023, we conducted cross-sectional PPFP needs assessments in twelve rural government health facilities (two hospitals, four health centers, six health posts) and focus group discussions (FGDs) among couples attending antenatal clinics to understand women's and men's perceptions and barriers to PPFP uptake.

Results: Seventeen (65%) hospital nurses and 11 (23%) health center nurses were trained in implant insertion, and 6 (23%) hospital nurses and 4 (9%) health center nurses were trained in postpartum IUD (PPIUD) insertion. Hospitals provided an average of 204 postpartum implants (29% per delivery) and 7 PPIUDs per month (1% per delivery), while health centers provided 25 postpartum implants and no PPIUDs per month. At health posts, there was no equipment for implant or IUD provision. The majority of the 27 couples recruited for FGDs (93% of women, 74% of men) had received family planning educational counseling though less than half (41%) of couples reported learning about the PPIUD. Participants reported concerns about the lack of information on how family planning works and side effects.

Conclusions: Our study highlights the need to enhance PPFP training and provision in rural areas, especially for PPIUD, and confirmed a low postpartum LARC uptake. Knowledge gaps and concerns about side effects were emphasized in FGDs. PPFP demand creation strategies tailored

for the rural populace as well as provider training could improve PPFP uptake in rural government clinics of Rwanda. Rural health posts could be explored as potential locations for PPFP counseling.

Keywords: *Postpartum; contraception; family planning; couples; assessment of health care needs; Rwanda; intrauterine device*

Background

The World Health Organization (WHO) recommends postpartum family planning (PPFP) for birth spacing, prevention of unintended pregnancy and abortion, and maternal-child health (1-3). PPFP positively impacts women, children, and nations. Short birth spacing intervals are associated with preterm birth, low birth weight, small gestational age, and maternal-child death (2, 4). It is estimated that PPFP reduces annual maternal deaths by 30% and child deaths by 10% in developing countries by preventing unintended pregnancy and spacing pregnancies ≥2 years as recommended by the WHO (2, 5-7).

Long-acting reversible contraceptives (LARC) are highly effective, cost-effective, feasible, acceptable (8-11), and safely used postpartum (1, 2). LARCs include the copper intrauterine device (IUD) and implant which last >12 and >5 years and cost $0.40 and $9 USD (12), respectively. In contrast, shorter-acting pills and injectables have lower effectiveness (2) and are refilled monthly or 3-monthly at a cost of $1 USD/cycle (12).

PPFP and LARC are prioritized in the Rwanda Family Planning 2030 Commitment (13, 14). However, similar to other countries in the region, 51% of married Rwandan women have unmet PPFP need, and this unmet need is more than two times higher compare to non-postpartum periods (15, 16). LARC use also remains low: 16.5% of Rwandan women use implants and 1.2% use IUDs (16).

PPFP could have a major life-saving impact for rural Rwandans. Most (80%) Rwandans live in rural areas (16). Though desires for large family sizes in rural areas have decreased due to government

educational campaigns on health, poverty, and land scarcity (17), rural total fertility remains higher compared to urban areas (4.3 vs 3.4) despite the mean desired number of children being similar to urban areas (3.5 vs 3.4). Though rural areas have seen a 39% increase in family planning use over the past decade (16, 18), rural women report lower IUD use (0.7% vs 3%) than urban women (16). Additionally, inter-pregnancy intervals are shorter and rates of infant, under-5 (16), and maternal (19) mortality are higher in rural vs urban women.

Rural women/couples face distinct challenges and opportunities to access PPFP. Challenges include more poverty and financial healthcare access barriers, lower education and literacy, and longer distances to health facilities (16). A recent study found travel distance made it difficult for rural Rwandan women to attend all four recommended antenatal care (ANC) visits (20), and rural women have concerns about transportation costs (21). Studies conducted in Rwanda (22-25) and other countries across the region (26, 27) show limited male partner involvement in family planning counseling. Similar to women living in urban areas, most (88%) rural women report family planning is a decision between husband and wife (16), though engaging men can be challenging. In our prior study in 30 rural Rwandan health centers, 77% of facility directors and family planning providers expressed concerns about engaging men in family planning decisions due to men not being willing to wait at the facility and miss income opportunities ((28).

Opportunities to access PPFP include the Rwanda's unique healthcare system. A robust community health worker (CHW) network provides FP and PPFP in promotion in the community, and CHW accompany and refer clients to health centers or health posts and provide short-term

methods after initiation at a health facility. Health posts are Rwanda's most basic healthcare facilities and are overseen by health centers in the same catchment area. Health posts provide a limited range of services such as outpatient care, child immunizations, growth monitoring for children under the age of five, antenatal care, family planning counseling and provision, health education, and few laboratory tests (Malaria rapid test, HIV testing, Pregnancy test, Gylcemie/Glucosurie rapid test). Health posts refer patients to health centers, and in some cases, a district hospital. Health centers provide family planning counseling and provision, preventive and curative healthcare, and referrals to district hospitals if necessary. Most health centers have nurses and midwives but not doctors (except for medicalized health centers). Hospitals do not provide ANC or infant vaccination but do provide all curative health services based on their level, with district hospitals, provincial referral hospitals, and national referral teaching hospitals offering FP and PPFP counseling as well as short-term, long-term, and permanent techniques ((28).

In this mixed methods study, we assessed rural health facility resources and gaps for PPFP provision, and we conducted focus group discussions (FGDs) with couples to understand PPFP knowledge and barriers to uptake.

Methods

Study design

This study used mixed methods including quantitative data from a cross-sectional needs assessment survey and qualitative data from the FGDs.

Study sites and recruitment

Study preparation began in May 2023, and the participant recruitment period was from July 10-19, 2023 in two rural districts in Rwanda, Rusizi and Rubavu. High-volume and accessible health facilities were selected for PPFP needs assessment and FGDs. Twelve facilities were selected: one district hospital, two affiliated health centers, and three health posts per district.

FGDs were recruited from ANC clinics within the four selected health centers (only health centers conduct routine ANC visits). Eligible couples were pregnant women attending ANC visits with their partners, at least 18 years of age, willing to participate as couples in the FGDs, and provided signed informed consent prior to FGDs.

Quantitative data collection and analysis: PPFP needs assessments

To understand the current status of PPFP service delivery in the selected rural facilities, we conducted needs assessments using a standardized questionnaire to assess facility volume in different maternal-child services, the human resource capacity to provide PPFP services, and whether facilities had materials available for PPFP educational counseling and service provision. For the facility personnel, we recorded the number of available and trained providers (nurses, midwives, and physicians) in PPFP promotion and provision, and the number of affiliated and trained community health workers (CHW) in PPFP/Postpartum Long Acting Reversible Contraception (PPLARC) promotion in the community (28). We also extracted facility-level data

over six months (November 2022-April 2023) from monthly reports: the facility catchment population and monthly numbers of women attending ANC, delivery, and PPFP services; and type of PPFP provided.

The needs assessment questionnaire was administered by the Center for Family Health Research (CFHR) interview team, consisting of two trained nurses and one intern student. The team first met with each facility's key stakeholders, including the facility director, heads of departments, and the data manager. Interviews were conducted in Kinyarwanda and translated into English.

No personal identifiers were collected. Needs assessment data were entered into a Microsoft Access database, cleaned, and analyzed descriptively using Excel.

Qualitative data collection and analysis: FGDs

We used a standardized focus group guide developed in English based on prior studies, predefined priority areas, and observation of PPFP services on the ground. Topics included current knowledge about PPFP/PPLARC, barriers and facilitators to better access and uptake PPFP, the role of male involvement in PPFP uptake and strategies to engage men more, PPFP educational tool feedback, and the importance of peer testimonials strategy during PPFP uptake. The final guide was translated into Kinyarwanda and pilot-tested.

The FGDs were conducted in July 2023 at the selected rural health centers by two trained CFHR nurses, with one nurse moderating the FGD and the other taking notes. These discussions were also audio-recorded to avoid missing any participant views during the transcription process. We conducted six FGDs with 6-12 participants per session with a time duration ranging between 1

hour 45 minutes to 2 hours 15 minutes with 3 FGDs in each hospital catchment area. After completion of an FGD, notes/recordings were translated from Kinyarwanda to English by CFHR nurses, and the transcripts were imported into NVivo software to create a list of preliminary themes using the participants' own words.

Ethics

We used de-identified programmatic data shared by the government facility's key stakeholders during the needs assessments. Ethical approval to conduct FGDs was obtained from the Rwanda National Ethics Committee and Emory IRB, and written informed consent from eligible couples was obtained before allowing them to participate in any FGD and each participant was compensated for the time.

Results

Human resources and equipment for PPFP

The two district hospitals served a catchment population size of 737,233 across 731 villages while the four affiliated health centers served a subset catchment population size of 186,508 across 172 villages (**Table 1**). A total of 2,808 CHW were affiliated with hospitals of whom 571 (20%) were in charge of pregnant women and newborn health. Another 633 CHW were affiliated with the health centers of whom 26% were in charge of pregnant women and newborn health, 14% were in charge of family planning, 59% were trained in PPFP promotion, and 51% were trained in PPLARC promotion.

Table 1. Human resources and equipment for PPFP in rural-serving health facilities										
	Hospitals				Health Centers					
	Total		Gisenyi	Gihundwe	Total		Kigufi	Rugerero	Bugarama	Giheke
	N	%	N	N	N	%	N	N	N	N
Catchment population	737,233	–	546,683	190,550	186,508	–	48,718	66,926	49,207	21,657
Villages served	731	–	518	213	172	–	48	51	37	36
Total CHW	2,808	–	1,990	818	663	–	192	179	148	144
In charge of pregnant women and newborn health	571	20%	358	213	172	26%	48	51	37	36
In charge of family planning	–	–	–	–	94	14%	46	33	9	6
Trained in PPFP promotion	–	–	–	–	388	59%	96	0	148	144
Trained in PPFP IUD/implant promotion	–	–	–	–	338	51%	46	0	148	144
Total nurses	206	–	121	85	47	–	10	16	12	9
Total nurses in maternity* department at hospitals	26	–	17	9	47	–	10	16	12	9
Trained in PPFP promotions	17	65%	10	7	17	36%	1	3	2	5
Trained in IUD insertion	7	27%	4	3	7	15%	1	2	2	2
Trained in PPIUD insertion	6	23%	4	2	4	9%	1	0	2	1
Trained in implant insertion	17	65%	10	7	11	23%	1	3	2	5
Total midwives	47	–	29	18	5	–	1	1	1	2
Assigned in maternity	44	–	29	15	5	–	1	1	1	2
Trained in PPFP promotions	36	82%	29	7	2	40%	1	0	0	1
Trained in IUD insertion	7	16%	2	5	2	40%	1	0	0	1
Trained in PPIUD insertion	7	16%	2	5	2	40%	1	0	0	1
Trained in implant insertion	34	77%	29	5	2	40%	1	0	0	1
Total physicians in maternity department	22	–	10	12	–	–	–	–	–	–
Trained in PPFP promotion	7	32%	4	3	–	–	–	–	–	–
Trained in IUD insertion	7	32%	4	3	–	–	–	–	–	–
Trained in PPIUD insertion	7	32%	4	3	–	–	–	–	–	–
Trained in implant insertion	3	14%	0	3	–	–	–	–	–	–
Trained in intra-cesarean PPIUD insertion	7	32%	4	3	–	–	–	–	–	–
Equipment amounts available										
IUD methods	235	–	220	15	106	–	23	32	36	15
Implants methods	379	–	322	57	187	–	70	95	14	8
Disposable Jadelle kits	333	–	322	11	184	–	70	95	11	8
Non-disposable Jadelle kits	0	–	0	0	0	–	0	0	0	0
Forceps	50	–	6	44	47	–	3	14	9	21
Speculum	21	–	6	15	37	–	6	4	10	17
Sims vaginal speculum	17	–	4	13	13	–	1	6	6	0
Hysterometer	11	–	7	4	8	–	2	5	1	0
Tenaculum forceps	11	–	4	7	9	–	3	2	3	1

Kelly forceps	12	–	3	9	17	–	1	8	4	4
Gynecological tables	13	–	8	5	10	–	2	3	2	3
Gynecological lamps	3	–	2	1	2	–	1	0	0	1
String scissors	17	–	14	3	8	–	1	3	2	2
IUD/Implants kits able to be sterilized per day	30	–	18	12	26	–	10	12	2	2
Equipment presence										
Working generator	–	–	Yes	Yes	–	–	Yes	No	No	No
Autoclave	–	–	Yes	Yes	–	–	Yes	Yes	No	Yes
Dry heat sterilization	–	–	No	No	–	–	Yes	Yes	Yes	No
Television	–	–	Yes	Yes	–	–	Yes	No	No	No
Family planning educational counseling materials	–	–	Yes	Yes	–	–	Yes	Yes	Yes	Yes
NGO or other partner in PPFP or PPIUD	–	–	No	Yes	–	–	No	No	Yes	No

CHW: Community health workers

NGO: non-governmental organization

PPIUD: postpartum intrauterine device

IUD: intrauterine device

PPFP: postpartum family planning

*Or ANC for health centers

182

Of the 206 nurses working at hospitals, 26 were assigned to maternity departments, of whom 65% were trained in PPFP promotions, 27% were trained in IUD insertion, 23% were trained in PPIUD insertion, and 65% were trained in implant insertion. Among 44 midwives working at hospitals and assigned in maternity departments, 82% were trained in PPFP promotion, 16% were trained in IUD and PPIUD insertion, and 77% were trained in implant insertions. Of the 47 nurses working at health centers, 36% were trained in PPFP promotions, 15% were trained in IUD insertion, 9% were trained in PPIUD insertion, and 23% were trained in implant insertion. Of 5 midwives at health centers, 40% were trained in PPFP promotion, IUD, PPIUD, and implant insertion. Only hospitals employed physicians; of 22 physicians working in maternity department, 32% were trained in PPFP promotion, IUD insertion, PPIUD insertion (including intra-cesarean PPIUD insertion), and 14% were trained in implant insertion.

All six facilities reported available LARC methods in stock, implant disposable insertion kits, equipment for LARC insertion (forceps, specula, and gynecological exam tables), the capacity to sterilize IUD insertion kits either by an autoclave or dry heat sterilization, and family planning educational counseling materials. However, few gynecologic lamps were available and only half of the health centers had a working television and generator.

PPLARC provision

The two hospitals performed an average of 704 deliveries per month and provided 204 PP implants, 7 PPIUD, and 3 PPIUD follow-up appointments (**Table 2**). Almost one-third (30%) of

women delivering at hospitals received a PPLARC (29% implant, 1% IUD). The four health centers performed 145 deliveries per month and provided 25 PP implants and no PPIUDs. Almost one-fifth (17%) of women delivering at health centers received a PPLARC (17% implant, 0% IUD).

Table 2. Monthly average service volume and PPLARC provision from November 2022 to April 2023 in rural-serving health facilities

	**Hospitals**			**Health Centers**				
	**Total**	**Gihundwe**	**Gisenyi**	**Total**	**Kigufi**	**Rugerero**	**Bugarama**	**Giheke**
	**N**	**N**	**N**	**N**	**N**	**N**	**N**	**N**
Facility service attendees								
Infant vaccination attendees*	–	–	–	2058	638	472	690	258
Antenatal care attendees*	–	–	–	320	88	83	100	49
Deliveries	704	224	480	145	24	35	65	21
Method provision								
PPLARC total	210	39	171	25	7	11	6	2
Postpartum implant insertions	204	36	168	25	7	11	6	2
PPIUD insertions	7	4	3	0	0	0	0	0
PPIUD Follow Up	3	3	0	0	0	0	0	0
Interval (non-postpartum) IUD insertions	11	6	5	5	1	2	0	3
	**%**	**%**	**%**	**%**	**%**	**%**	**%**	**%**
PPLARC insertions per delivery	30%	18%	36%	17%	28%	30%	8%	8%
Postpartum implant insertions per delivery	29%	16%	35%	17%	28%	30%	8%	8%
PPIUD insertions per delivery	1%	2%	1%	0%	0%	0%	0%	0%

*Services provided at health centers only

PPIUD: postpartum intrauterine device

PPLARC: postpartum long-acting reversible contraception

IUD: intrauterine device

Among the six health posts, only one was providing delivery services with an average of 18 deliveries monthly (data not tabled). There was no available equipment to facilitate implant and IUD provision, and no PPLARC provision was performed. All health posts were only providing short-term contraceptive methods.

Couple focus group discussions

We conducted six FGDs including 27 couples (**Table 3**). The average age for women was 28 years, ranging from 20 to 43 years, while the average age for men was 31 years, ranging from 21 to 58. The average number of pregnancies including the current pregnancy was three, with a range of 1 to 7. 63% of women and 85% of men had completed primary education only, and an additional 15% of women did not complete any education.

Table 3. Characteristics of focus group participants (N=27 pregnant couples)

	Women		Men	
	**Mean**	**Range**	**Mean**	**Range**
Age, years	28	20-43	31	21-58
Number of pregnancies	3	1–7	--	--
	**N**	**%**	**N**	**%**
Education level				
None	4	15%	0	0%
Primary	17	63%	23	85%
High school	6	22%	4	15%
Received FP education/counseling*	25	93%	20	74%
Discussed short-term methods	22	81%	16	59%
Discussed implants	22	81%	13	48%
Discussed IUD	5	19%	2	7%
Received PPFP education/counseling	23	85%	17	63%
Know importance of PPFP	26	96%	19	70%
Women with concerns about side effects	16	60%	--	--
Concerns about cost	4	16%	--	--
Add cost and tim	6	22%	--	--
Religion barrier	9	33%	--	--
Strategies to encourage women to consider or obtain PPFP				
Educated the couple together	3	11%	--	--
Involvement of male	11	41%	--	--

*17 couples (63%) received joint FP counseling/education; providers suggested use of implant to 20 couples (74%) and use of PPIUD to 11 (41%) couples

IUD: intrauterine device

PPFP: postpartum family planning

The majority (93%) of women had received previous family planning educational counseling in a health center, whereas nearly three-fourths (74%) of men reported having had previous family planning educational counseling in a health center in ANC. Men and women who had not received family planning educational counseling in a health center reported hearing about family planning on the radio. Almost two-thirds (63%) of couples had received family planning educational counseling together, primarily in ANC, and one couple reported receiving family planning education from a CHW during a village meeting. The majority of women who had received family planning education had heard about implants and short-term methods (81%), but few (19%) had received education about the IUD.

Most (85%) female participants had received PPFP education at health facilities after delivery, and one female participant said that she had heard for the first time about PPFP during her most recent delivery despite previous health facility deliveries. The majority of female participants (96%) thought PPFP was important, and one said that "**PPFP assists women in recovering and gaining the ability to work, contribute to family income, and alleviate poverty. It aids in birth spacing and gives women confidence in preventing unwanted pregnancy."** Fewer male participants (63%) had received PPFP education or counseling; however, most (70%) were aware of the importance of PPFP: "**The wife can conceive a few months after giving birth, and if she uses the PPFP methods, there is no concern of an unintended pregnancy.**"

The most commonly suggested PPFP methods by providers were pills, injectables, and implants. For 74% of couples, PP implants were an option that was suggested by providers, and couples

knew why it was important to uptake PP implants after delivery. For example, "**It prevents a woman from becoming pregnant while her child is still young and permits her to become pregnant when the youngest has grown up; it is easy to be removed; there are 2 options for 3 and 5 years; it helps in child spacing; and it helps for the wellbeing of the mother and the child."** One participant who did not receive PPFP educational counseling said, "**No, I had never heard about PP implants from a health professional, but I did read or get some information on them, such as that if a woman chooses PP implants and experiences negative effects, she may return to the health center and switch to another procedure**.**"** Only 41% of couples said that PPIUD was an option that was discussed with them by a provider. One participant stated about the PPIUD: "**I just know it exists because some of my friends use it", another participant said that** " **I had never heard of PPIUD except from reading about it on hospital posters and hearing about it on the radio, and I had never discussed it with anyone who used it."**

Some men asked questions about how IUDs and implants work so that they could explain them to their wives. One man asked, "**You said that there is a PPIUD; explain to us how it can be used. Is it like other methods, like pills or injectables? "**, "**And I also need more explanation on PPIUD."** More generally, another participant said, **"We are mostly concerned with a lack of insufficient information on how family planning methods work."** Other participants raised concerns about side effects and rumors heard from the community: **"I heard that the implant can move and go to other places of the body, but I'm not sure if that can harm the woman."** and "**I would like to know if this is true; when someone uses an implant and has amenorrhea, can this cause tumors? I was using an implant and removed it due to fear of developing**

tumors.” Some participants (7%) reported that the IUD bothers the male partner during sexual intercourse.

Some participants (60%) expressed concern about the side effects of the implant, including bleeding, dizziness, weight loss or gain, backache and headache, and genital infections. One participant said that **“sometimes FP methods cause side effects that prevent people from taking them in the future."** Some men (16%) expressed concern about the potentially high cost spent on medical management of side effects.

Additional cost and time concerns were also expressed by 22% of participants. One participant stated, **“It takes our time. It is not easy to convince a provider to remove it when you want to. Sometimes, it requires going to a private clinic for removal, which is expensive,”** and **“there is also a lack of health insurance in some families, which prevents them from taking FP.”** Another participant noted that **“it is free to access family planning services at the health center immediately after delivery; there is not much time spent receiving family planning methods. The problem is when you want to remove the family planning method.”**

Religion was occasionally (33%) expressed as a barrier: “**Most religious people do not support family planning; they said, this is to kill children inside the womb, and we are asking ourselves how we killed someone who does not yet exist. They promote traditional methods, which are not sins. People decided to take a risk and use the modern family planning method. Using the family planning method is regarded as a sin (killing humans), which contradicts God's advice:**

"Multiply your descendants until you become as numerous as the sands on the sea"." Additionally, two male participants said that some people think that family planning is for poor families who cannot afford to take care of their children.

Participants suggested receiving comprehensive explanations about family planning methods before provision: "**The provider must explain the advantages and disadvantages of each family planning method before providing it. This may help the client choose the method when well aware of its side effects."** Another suggested strategy was to start the family planning conversation early during pregnancy: "**Family planning discussions should start in the family before coming to the health facility in order to avoid making bad decisions at the time of provision after delivery. Discussing early on would allow them to check through the pros and cons of family planning methods and reach the facility ready to uptake."**

Some (11%) participants suggested PPFP education be provided to couples and that men could be approached for family planning education by sending a CHW to invite the couple to receive PPFP education together at home, during community gatherings, at church, during ANC visits, or at delivery. Involving male partners was viewed by most (41%) as very important. Participants said men should have a significant role in family planning decisions: "**Men need to understand family planning in order to support their wives; they have power or influence during the decision-making process of family planning; once they are against family planning, it is a challenge; and what is missing are promotional talks; and male partners are not exposed enough to health promotion. I am confident that men understand better once educated, and**

the decisions of family planning come from both partners. Discussing early on would allow them to check through the pros and cons of family planning methods and reach the facility ready to uptake." One participant noted that "**one partner refuses to proceed alone with getting the PPFP method, or when the woman takes the PPFP without husband approval, when side effects come, it creates misunderstanding in the couple."**

All participants thought it would be helpful to receive testimonials from peers or women who are satisfied with their PPFP methods; one woman noted that "**listening to happy clients who talk about the positive impact of using PPFP would encourage people to uptake these methods**" and **"hearing testimonials reinforce hope and influence family planning decisions."** One participant who received such a testimonial previously said that "**it would be helpful, and when I was a single, a woman who had planned successfully another pregnancy after nine years inspired me to use PPFP."** However, there was one participant who noted: "**If I hear testimonials from unhappy clients, then I wouldn't use them at all, and I would discourage others from using them as well."**

Discussion

According to the *Scaling Up Immediate Postpartum Family Planning Services in Rwanda* report (29), the rollout and extension of PPFP services began in 2016 with capacity building in selected districts and scaling in all districts of Rwanda by 2020, and the availability and accessibility of PPFP services at all levels have increased. Our study found that PPFP services are being offered in rural health centers and hospitals, though few nurses and midwives are trained in PPIUD provision with physicians at hospitals trained in PPIUD insertion (32%) more than implant

insertion (14%). Over half of CHW had received training in PPFP promotion. Not all essential materials to facilitate the provision of PPLARC methods were available at the health centers and hospitals, few nurses were trained in LARC provision, and there was a lack of PPFP provision materials, similar to our previous work in 2016 conducted in 30 rural health settings in Rwanda (28).

In terms of PPLARC method provision, we found that all health facilities were providing PP implants, but no PPIUD methods were provided at Health centers despite available stock for any FP methods, similar to another study that showed that the most commonly provided LARC method in Rwanda was implants and FP initiators prefer implants (28). Low PPIUD provision can be due to limited provider training, lack of effective counseling, lack of client knowledge on PPIUD, and misinformation. PPIUD is the most effective and cost-effective method for preventing pregnancy with few side effects compared to implants (8-11).

The findings of this study showed that most pregnant couples had received family planning and PPFP education and counseling, and they knew the importance of FP and PPFP. This is largely due to family planning and PPFP promotion during antenatal services at health centers, and in Rwanda, at the first ANC service, both partners attended the ANC visit together and received PPFP education. Between 2010 and 2020, there was an increase in modern family planning use in Rwanda, which is attributed to changes in knowledge and attitudes toward family planning among rural women (30). Sustained family planning use in rural Rwandan women has been associated with an increase in patient-centered counseling on potential side effects and peer counseling (30).

This study found that there is a lack of knowledge on how the family planning methods work and their side effects, but there is an opportunity to increase family planning and PPFP education or counseling in rural health facilities, as most rural women attend at least one ANC visit (98%), deliver in a health facility (91%), and obtain all eight basic infant vaccinations (95%), making these highly viable service delivery points for PPFP promotion and provision (16).

Most of the couples report that the PP implant was the method most discussed with them by providers, and this may be due to the smaller number of providers who are trained to provide the PPIUD method. The participants of this study reported that male partners have a significant role in PPFP method uptake decisions; female partners may refuse to uptake the PPFP method when male partners are absent without prior discussion. Our PPFP work in Kigali found male partners can be engaged by delivering educational counseling materials designed for couples at first ANC visits, 80% of which are attended by male partners in Rwanda (22, 31-34). Health posts are the primary healthcare closest to the community and findings of this study have shown that there is a high need to increase the capacity and training of healthcare providers for FP and PPFP promotion and provision.

The ongoing PPFP intervention (Icyemezo Cyacu, 'Our Decision' in Kinyarwanda) which was informed by women and their male partners, providers, and stakeholders will guide in the sustainability and cost-effectiveness of PPFP in urban areas (35, 36). The focus group participants requested FP education for couples to increase uptake which is similar to a previous study showing that integration of FP counseling in couples HIV counselling and testing increases uptake of LARC methods (35, 36).

Conclusion

The Rwandan government and its partners have put efforts into FP and PPFP accessibility and availability of all FP methods, which has resulted in increased FP uptake at all levels. To reach Rwanda's FP2030 commitment for expansion of access to FP for women and girls throughout the country and to reduce the unmet need for FP/PPFP, continuing to engage all healthcare systems from health posts to hospitals in rural and urban areas is critical to achieving the set target.

References:

1. WHO. Medical eligibility criteria for contraceptive use Geneva: WHO Press; 2015 [cited 2018 August 3]; 5th:[Available from: https://iris.who.int/bitstream/handle/10665/172915/WHO_RHR_15.07_eng.pdf?sequence=1

2. World Health Organization. Programming Strategies for Postpartum Family Planning. Geneva, Switzerland2013.

3. World Health Organization. Report of a WHO technical consultation on birth spacing (13-15 June 2005). Geneva, Switzerland 2007.

4. DaVanzo J, Hale L, Razzaque A, Rahman M. Effects of interpregnancy interval and outcome of the preceding pregnancy on pregnancy outcomes in Matlab, Bangladesh. BJOG : an international journal of obstetrics and gynaecology. 2007;114(9):1079-87. Epub 2007/07/10. doi: 10.1111/j.1471-0528.2007.01338.x. PubMed PMID: 17617195; PMCID: PMC2366022.

5. Darroch J, AAudam S, Biddlecom A, al. e. Adding It Up: In-vesting in Contraception and Maternal and Newborn Health. New York: Guttmacher Institute, 2017.

6. Cleland J, Bernstein S, Ezeh A, Faundes A, Glasier A, Innis J. Family planning: the unfinished agenda. The Lancet. 2006;368(9549):1810-27. doi: 10.1016/S0140-6736(06)69480-4.

7. Rutstein SO. Effects of preceding birth intervals on neonatal, infant and under-five years mortality and nutritional status in developing countries: evidence from the demographic and health surveys. Int J Gynaecol Obstet. 2005;89 Suppl 1:S7-24. Epub 2005/04/12. doi: 10.1016/j.ijgo.2004.11.012. PubMed PMID: 15820369.

8. FHI360. Assessing the Feasibility of Postpartum IUD Provision in Rwanda 2013 [cited 2016 May 5]. Available from: http://www.fhi360.org/sites/default/files/media/documents/assessing-feasibility-postpartum-iucd-rwanda-2013.pdf.

9. USAID, MCHIP, PSI. PPIUD Services: Start-Up to Scale-Up Regional Meeting Burkina Faso 2014 [cited 2018 August 3]. Available from: https://pdf.usaid.gov/pdf_docs/PA00JXWK.pdf.

10. Cwiak C, Cordes S. Postpartum intrauterine device placement: a patient-friendly option. Contraception and reproductive medicine. 2018;3:3. Epub 2018/04/25. doi: 10.1186/s40834-018-0057-x. PubMed PMID: 29686886; PMCID: PMC5899832.

11. PSI, USAID. Enabling the Healthy Spacing and Limiting of Pregnancies: Programmatic Approaches to Expand Postpartum IUD 2015 [cited 2019 May 21]. Available from: https://pdf.usaid.gov/pdf_docs/PBAAD694.pdf

12. Management Sciences for Health. International Medical Products Price Guide 2016 [cited 2020 June 27]. Available from: https://mshpriceguide.org/en/home/.

13. STATCompiler: The DHS Program. Rwanda DHS 2019-2020 [cited 2023 May 1]. Available from: https://www.statcompiler.com/en/.

14. Muhoza DN. The heterogeneous effects of socioeconomic and cultural factors on fertility preferences: evidence from Rwanda and Kenya. Journal of Population Research. 2019;36(4):347-63.

15. Kalinda C, Phiri M, Chimpinde K, Ishimwe MCS, Simona SJ. Trends and socio-demographic components of modern contraceptive use among sexually active women in Rwanda: a multivariate decomposition analysis. Reprod Health. 2022;19(1):226. Epub 2022/12/17. doi: 10.1186/s12978-022-01545-0. PubMed PMID: 36527042; PMCID: PMC9758849.

16. National Institute of Statistics of Rwanda (NISR). Mortality Assessment Survey 2015 [cited 2023 May 1]. Available from: https://www.statistics.gov.rw/publication/mortality-assessment-survey-mas-2015.

17. Dusingizimana T, Ramilan T, Weber JL, Iversen PO, Mugabowindekwe M, Ahishakiye J, Brough L. Predictors for achieving adequate antenatal care visits during pregnancy: a cross-sectional study in rural Northwest Rwanda. BMC pregnancy and childbirth. 2023;23(1):69. Epub 2023/01/27. doi: 10.1186/s12884-023-05384-0. PubMed PMID: 36703102; PMCID: PMC9878946.

18. Adler AJ, Randall T, Schwartz LN, Drown L, Matthews S, Pace LE, Mugabo C, Kateera F, Bukhman G, Baganizi E, Ng'ang'a LM. What women want: A mixed-methods study of women's health priorities, preferences, and experiences in care in three Rwandan rural districts. Int J Gynaecol Obstet. 2023. Epub 2023/02/24. doi: 10.1002/ijgo.14735. PubMed PMID: 36815725.

19. Da Costa V, Ingabire R, Sinabamenye R, Karita E, Umutoni V, Hoagland A, Allen S, Mork E, Parker R, Mukamuyango J, Haddad L, Nyombayire J, Wall KM. An Exploratory Analysis of Factors Associated With Interest in Postpartum Intrauterine Device Uptake Among Pregnant Women and Couples in Kigali, Rwanda. Clinical medicine insights Reproductive health. 2019;13:1179558119886843. Epub 2019/12/17. doi: 10.1177/1179558119886843. PubMed PMID: 31839717; PMCID: PMC6893932.

20. Haddad L, Wall KM, Vwalika B, Khu NH, Brill I, Kilembe W, Stephenson R, Chomba E, Vwalika C, Tichacek A, Allen S. Contraceptive discontinuation and switching among couples receiving integrated HIV and family planning services in Lusaka, Zambia. Aids. 2013;27 Suppl

1:S93-103. Epub 2013/10/23. doi: 10.1097/QAD.0000000000000039. PubMed PMID: 24088689; PMCID: PMC4070372.

21. Mukamuyango J, Ingabire R, Parker R, Nyombayire J, Easter SR, Wall KM, Tichacek A, Nyirazinyoye L, Kaslow N, Allen S, Karita E. Motivational interviewing to promote long-acting reversible contraception among Rwandan couples wishing to prevent or delay pregnancy. American journal of obstetrics and gynecology. 2020;222(4s):S919.e1-S.e12. Epub 2019/12/16. doi: 10.1016/j.ajog.2019.11.1280. PubMed PMID: 31838122; PMCID: PMC7138747.

22. Khu NH, Vwalika B, Karita E, Kilembe W, Bayingana RA, Sitrin D, Roeber-Rice H, Learner E, Tichacek AC, Haddad LB, Wall KM, Chomba EN, Allen SA. Fertility goal-based counseling increases contraceptive implant and IUD use in HIV-discordant couples in Rwanda and Zambia. Contraception. 2013;88(1):74-82. Epub 2012/11/17. doi: 10.1016/j.contraception.2012.10.004. PubMed PMID: 23153896; PMCID: PMC3625675.

23. Ongolly FK, Bukachi SA. Barriers to men's involvement in antenatal and postnatal care in Butula, western Kenya. Afr J Prim Health Care Fam Med. 2019;11(1):e1-e7. Epub 2019/08/02. doi: 10.4102/phcfm.v11i1.1911. PubMed PMID: 31368318; PMCID: PMC6676928.

24. Tran NT, Yameogo WME, Gaffield ME, Langwana F, Kiarie J, Kulimba DM, Kouanda S. Postpartum family-planning barriers and catalysts in Burkina Faso and the Democratic Republic of Congo: a multiperspective study. Open Access J Contracept. 2018;9:63-74. doi: 10.2147/OAJC.S170150. PubMed PMID: 30519124.

25. Mazzei A, Ingabire R, Karita E, Mukamuyango J, Nyombayire J, Parker R, Tichacek A, Allen S, Wall KM. Cross-sectional assessment of government health center needs to implement long-acting reversible contraception services in rural Rwanda. BMC Womens Health. 2021;21(1):411.

Epub 2021/12/17. doi: 10.1186/s12905-021-01555-3. PubMed PMID: 34911535; PMCID: PMC8672509.

26.https://www.moh.gov.rw/fileadmin/user_upload/Moh/Publications/Legal_Framework/Public_health_Facilities_service_packages_in_Rwanda-1.pdf

27. Williams P, Morales K, Sridharan V *et al.* Postpartum family planning in Rwanda: a cost effectiveness analysis [version 3; peer review: 3 not approved]. *Gates Open Res* 2019, **3**:887 (https://doi.org/10.12688/gatesopenres.12934.3).

29. Schwandt H, Boulware A, Corey J, Herrera A, Hudler E, Imbabazi C, King I, Linus J, Manzi I, Merritt M, Mezier L, Miller A, Morris H, Musemakweli D, Musekura U, Mutuyimana D, Ntakarutimana C, Patel N, Scanteianu A, Shemeza BE, Sterling-Donaldson G, Umutoni C, Uwera L, Zeiler M, Feinberg S. Family planning providers and contraceptive users in Rwanda employ strategies to prevent discontinuation. BMC Womens Health. 2021;21(1):361. Epub 2021/10/13. doi: 10.1186/s12905-021-01503-1. PubMed PMID: 34635111; PMCID: PMC8504084.

30. Ingabire R, Nyombayire J, Hoagland A, Da Costa V, Mazzei A, Haddad L, Parker R, Sinabamenye R, Mukamuyango J, Smith J, Umutoni V, Mork E, Allen S, Karita E, Wall K. Evaluation of a multi-level intervention to improve post-partum intrauterine device services in Rwanda. Gates Open Research. 2018;2(38). doi: 10.12688/gatesopenres.12854.1. PMC6266741.

31. Tounkara MS, Ingabire R, Comeau DL, Karita E, Allen S, Nyombayire J, Parker R, Haddad LB, Da Costa V, Tichacek A, Mazzei A, Mukamuyango J, Wall KM. A mixed-methods study of factors influencing postpartum intrauterine device uptake after family planning counseling among women in Kigali, Rwanda. PLoS One. 2022;17(11):e0276193. Epub 2022/11/04. doi: 10.1371/journal.pone.0276193. PubMed PMID: 36327214; PMCID: PMC9632907.

32. Påfs J, Musafili A, Binder-Finnema P, Klingberg-Allvin M, Rulisa S, Essén B. 'They would never receive you without a husband': Paradoxical barriers to antenatal care scale-up in Rwanda. Midwifery. 2015;31(12):1149-56. doi: https://doi.org/10.1016/j.midw.2015.09.010.

33. Tuyisenge G, Hategeka C, Kasine Y, Luginaah I, Cechetto D, Rulisa S. Mothers' perceptions and experiences of using maternal health-care services in Rwanda. Women & health. 2019;59(1):68-84. Epub 2018/02/06. doi: 10.1080/03630242.2018.1434591. PubMed PMID: 29400640.

34. Wall K. Icyemezo Cyacu ('Our Decision'): Implementing postpartum family planning services in Kigali, Rwanda 2020 [cited 2023 Mar 1]. Available from: https://scholarblogs.emory.edu/rwandappfp/.

35. Mukamuyango J, Ingabire R, Parker R, Nyombayire J, Abaasa A, Asiki G, Easter SR, Wall KM, Nyirazinyoye L, Tichacek A, Kaslow N, Price MA, Allen S, Karita E. Uptake of long acting reversible contraception following integrated couples HIV and fertility goal-based family planning counselling in Catholic and non-Catholic, urban and rural government health centers in Kigali, Rwanda. Reprod Health. 2020 Aug 17;17(1):126. doi: 10.1186/s12978-020-00981-0. PMID: 32807177; PMCID: PMC7433361https://pubmed.ncbi.nlm.nih.gov/32807177/

1. WHO. Medical eligibility criteria for contraceptive use Geneva: WHO Press; 2015 [5th:[Available from: http://www.who.int/reproductivehealth/publications/family_planning/Ex-Summ-MEC-5/en/.

2. World Health Organization. Programming Strategies for Postpartum Family Planning. Geneva, Switzerland2013.

3. World Health Organization. Report of a WHO technical consultation on birth spacing (13-15 June 2005). Geneva, Switzerland 2007.

4. DaVanzo J, Hale L, Razzaque A, Rahman M. Effects of interpregnancy interval and outcome of the preceding pregnancy on pregnancy outcomes in Matlab, Bangladesh. BJOG : an international journal of obstetrics and gynaecology. 2007;114(9):1079-87.
5. Darroch J, AAudam S, Biddlecom A, al. e. Adding It Up: In-vesting in Contraception and Maternal and Newborn Health. New York: Guttmacher Institute; 2017.
6. Cleland J, Bernstein S, Ezeh A, Faundes A, Glasier A, Innis J. Family planning: the unfinished agenda. The Lancet. 2006;368(9549):1810-27.
7. Rutstein SO. Effects of preceding birth intervals on neonatal, infant and under-five years mortality and nutritional status in developing countries: evidence from the demographic and health surveys. Int J Gynaecol Obstet. 2005;89 Suppl 1:S7-24.
8. FHI360. Assessing the Feasibility of Postpartum IUD Provision in Rwanda 2013 [Available from: http://www.fhi360.org/sites/default/files/media/documents/assessing-feasibility-postpartum-iucd-rwanda-2013.pdf.
9. USAID, MCHIP, PSI. PPIUD Services: Start-Up to Scale-Up Regional Meeting Burkina Faso 2014 [Available from: https://www.psi.org/publication/piud-services-start-up-to-scale-up-regional-meeting-burkina-faso/.
10. Cwiak C, Cordes S. Postpartum intrauterine device placement: a patient-friendly option. Contraception and reproductive medicine. 2018;3:3.
11. PSI, USAID. Enabling the Healthy Spacing and Limiting of Pregnancies: Programmatic Approaches to Expand Postpartum IUD 2015 [Available from: https://www.psi.org/wp-content/uploads/2015/02/PPIUD_English-Web-a.pdf.
12. Management Sciences for Health. International Medical Products Price Guide 2016 [Available from: https://mshpriceguide.org/en/home/.
13. Family Planning 2020. Rwanda 2018 [Available from: http://www.familyplanning2020.org/entities/81.
14. Rwanda MOH. Family Planning 2020 Commitment 2017 [Available from: http://ec2-54-210-230-186.compute-1.amazonaws.com/wp-content/uploads/2018/02/Govt.-of-Rwanda-FP2020-Commitment-2018-Update.pdf.
15. USAID, MCHIP. Family planning needs during the first two years postpartum in Rwanda. 2010.
16. STATCompiler: The DHS Program. Rwanda DHS 2019-2020 [Available from: https://www.statcompiler.com/en/.
17. Muhoza DN. The heterogeneous effects of socioeconomic and cultural factors on fertility preferences: evidence from Rwanda and Kenya. Journal of Population Research. 2019;36(4):347-63.
18. Kalinda C, Phiri M, Chimpinde K, Ishimwe MCS, Simona SJ. Trends and socio-demographic components of modern contraceptive use among sexually active women in Rwanda: a multivariate decomposition analysis. Reprod Health. 2022;19(1):226.
19. National Institute of Statistics of Rwanda (NISR). Mortality Assessment Survey 2015 [Available from: file:///C:/Users/krist/Downloads/Mortality%20Assessment%20Survey_2015%20report%20(1).pdf.
20. Dusingizimana T, Ramilan T, Weber JL, Iversen PO, Mugabowindekwe M, Ahishakiye J, et al. Predictors for achieving adequate antenatal care visits during pregnancy: a cross-sectional study in rural Northwest Rwanda. BMC pregnancy and childbirth. 2023;23(1):69.
21. Adler AJ, Randall T, Schwartz LN, Drown L, Matthews S, Pace LE, et al. What women want: A mixed-methods study of women's health priorities, preferences, and experiences in care in three Rwandan rural districts. Int J Gynaecol Obstet. 2023.
22. Da Costa V, Ingabire R, Sinabamenye R, Karita E, Umutoni V, Hoagland A, et al. An Exploratory Analysis of Factors Associated With Interest in Postpartum Intrauterine Device Uptake Among Pregnant Women and Couples in Kigali, Rwanda. Clinical medicine insights Reproductive health. 2019;13:1179558119886843.

23. Haddad L, Wall KM, Vwalika B, Khu NH, Brill I, Kilembe W, et al. Contraceptive discontinuation and switching among couples receiving integrated HIV and family planning services in Lusaka, Zambia. Aids. 2013;27 Suppl 1:S93-103.
24. Khu NH, Vwalika B, Karita E, Kilembe W, Bayingana RA, Sitrin D, et al. Fertility goal-based counseling increases contraceptive implant and IUD use in HIV-discordant couples in Rwanda and Zambia. Contraception. 2013;88(1):74-82.
25. Mukamuyango J, Ingabire R, Parker R, Nyombayire J, Easter SR, Wall KM, et al. Motivational interviewing to promote long-acting reversible contraception among Rwandan couples wishing to prevent or delay pregnancy. American journal of obstetrics and gynecology. 2020;222(4s):S919.e1-S.e12.
26. Ongolly FK, Bukachi SA. Barriers to men's involvement in antenatal and postnatal care in Butula, western Kenya. Afr J Prim Health Care Fam Med. 2019;11(1):e1-e7.
27. Tran NT, Yameogo WME, Gaffield ME, Langwana F, Kiarie J, Kulimba DM, et al. Postpartum family-planning barriers and catalysts in Burkina Faso and the Democratic Republic of Congo: a multiperspective study. Open Access J Contracept. 2018;9:63-74.
28. Mazzei A, Ingabire R, Karita E, Mukamuyango J, Nyombayire J, Parker R, et al. Cross-sectional assessment of government health center needs to implement long-acting reversible contraception services in rural Rwanda. BMC Womens Health. 2021;21(1):411.
29. Maternal and Child Survival Program. Scaling Up Immediate Postpartum Family Planning Services in Rwanda 2018 [Available from: https://pdf.usaid.gov/pdf_docs/PA00TJWR.pdf.
30. Schwandt H, Boulware A, Corey J, Herrera A, Hudler E, Imbabazi C, et al. Family planning providers and contraceptive users in Rwanda employ strategies to prevent discontinuation. BMC Womens Health. 2021;21(1):361.
31. Ingabire R, Nyombayire J, Hoagland A, Da Costa V, Mazzei A, Haddad L, et al. Evaluation of a multi-level intervention to improve post-partum intrauterine device services in Rwanda. Gates Open Research. 2018;2(38).
32. Tounkara MS, Ingabire R, Comeau DL, Karita E, Allen S, Nyombayire J, et al. A mixed-methods study of factors influencing postpartum intrauterine device uptake after family planning counseling among women in Kigali, Rwanda. PloS one. 2022;17(11):e0276193.
33. Påfs J, Musafili A, Binder-Finnema P, Klingberg-Allvin M, Rulisa S, Essén B. 'They would never receive you without a husband': Paradoxical barriers to antenatal care scale-up in Rwanda. Midwifery. 2015;31(12):1149-56.
34. Tuyisenge G, Hategeka C, Kasine Y, Luginaah I, Cechetto D, Rulisa S. Mothers' perceptions and experiences of using maternal health-care services in Rwanda. Women & health. 2019;59(1):68-84.
35. Wall KM, Ingabire R, Mazzei A, Umuhoza C, Parker R, Tichacek A, et al. Implementation and evaluation of a large-scale postpartum family planning program in Rwanda: study protocol for a clinic-randomized controlled trial. Trials. 2022;23(1):337.
36. Wall K. Icyemezo Cyacu ('Our Decision'): Implementing postpartum family planning services in Kigali, Rwanda 2020 [Available from: https://scholarblogs.emory.edu/rwandappfp/.
