# Supplementary material for: Implementing postpartum family planning services in rural Rwanda: A mixed-methods study
Source: PLoS One. 2025 Dec 30;20(12):e0338627. doi: 10.1371/journal.pone.0338627 (PMC12752963; doi:10.1371/journal.pone.0338627)
Supplement: Attachment — Submitted filename: PONE-D-24-28650_R1_reviewer COMMENTS.pdf [file pone.0338627.s005.pdf]

This paper is interesting, but has a fair bit of revision necessary in order to be publishable.

As a minor point, there are too many writing and grammatical errors, particularly numerical inconsistencies (six instead of 6).

More substantively, it would be helpful to know more about these two regions of Rwanda. How to they compare to others in terms of population density, prior FP use, ethnic diversity (e.g. refugee populations from surrounding nations). Outside of Kigali much of the nation is rural, but there is still demographic diversity so more information on why these two regions are selected (beyond convenience) would be helpful to contextualize the results.

The quantitative analysis is somewhat limited in terms of comparisons. If some clinics/ hospitals/health posts have limited capabilities for PPFP, is that consistent with other regions? Of course 100% is ideal, but what is realistic, and how far are these numbers from the realistic goal?

Qualitative focus groups should include more information as to how data was collected. Were husbands and wives in the same room at the same time? Could this influence responses? It is a real strength of this paper to include male voices in family planning conversations, but much more depth and details is needed to explain the process through which this data was collected, and the potential sources of bias and error in a highly patriarchal culture.

Most importantly, what information are we gleaning from this study beyond the idea that more resources, training, and information would improve PPFP outcomes. What can we learn from this that would help Rwandan communities specifically and public health scholars more generally?
