# Supplementary material for: Implementing postpartum family planning services in rural Rwanda: A mixed-methods study
Source: PLoS One. 2025 Dec 30;20(12):e0338627. doi: 10.1371/journal.pone.0338627 (PMC12752963; doi:10.1371/journal.pone.0338627)
Supplement: Attachment — Submitted filename: PlosOne_ReviewerResponse_16May25.docx [file pone.0338627.s006.docx]

Reviewers' comments:

5. Review Comments to the Author

Please use the space provided to explain your answers to the questions above. You may also include additional comments for the author, including concerns about dual publication, research ethics, or publication ethics. (Please upload your review as an attachment if it exceeds 20,000 characters)

**Reviewer #1:** Thank you for the opportunity to review this manuscript. The study addresses an important topic concerning Postpartum Family Planning (PPFP) and utilizes both quantitative and qualitative data sources. However, the manuscript requires major revisions.

General comments
The methods and results sections need thorough revision, particularly regarding the qualitative study, which is poorly described. I recommend adhering to the COREQ guidelines to enhance the quality of the qualitative reporting. Additionally, please consult textbooks on mixed methods research, such as Creswell’s work, to provide readers with a better understanding of the different types of mixed methods studies and how to report them.
***Response: We appreciate these suggestions and have adhered to COREQ; the COREQ checklist is now included as an appendix. We have also expanded our qualitative data analysis methods to provide more details, and have revised our results to align with COREQ and guidance on concurrent mixed methods designs from Creswell & Plano Clark, 2011.***

Specific comments
\#### Abstract
- Methods: Clearly specify the analytical techniques used for the quantitative part of the study.

***Response: We now specify that the quantitative data were analyzed descriptively.***

- Results: Start by stating the total number of data points analyzed. Distinguish clearly between quantitative and qualitative results. While the abstract currently summarizes quantitative findings, please specify the key themes and main takeaways from the qualitative findings without including specific values.

***Response:* We now distinguish the qualitative and the quantitative data in the abstract, detail the total number of data points analyzed for each analysis, and summarize the key qualitative themes.**

\#### Background
Although this section provides necessary context, it lacks a rationale for the study's importance. Please revise to highlight research gaps and the implications of your findings, creating a narrative that connects to the study objectives.

The last paragraph of the introduction should clearly articulate the research objectives. It currently summarizes the methods rather than stating the study’s aims.

***Response: The last paragraph of the introduction was revised to highlight the gaps in current knowledge that this study addresses.***

\#### Methods
- **Study Design**: Specify the type of mixed methods used (sequential explanatory/exploratory or concurrent design). If sequential, indicate whether quantitative or qualitative data collection precedes the other. (Refer to Designing and Conducting Mixed Methods Research
Book by John W. Creswell and Vicki L. Plano Clark)

***Response: We have expanded our qualitative data analysis methods to provide more details, and have revised our results to align with COREQ and guidance on concurrent mixed methods designs from Creswell & Plano Clark, 2011.***

- **Study Sites and Recruitment**: Clarify how and who determined that the facilities were high-volume and accessible. What criteria were used? Was it based on annual patient flow, formal reports, or insights from key stakeholders?

***Response: We now clarify how facilities were selected for inclusion in the methods section.***

- **Sampling**: Specify the sampling methods for both quantitative and qualitative data collection. Was convenience sampling used for quantitative data or was it sourced from a database? Was purposive sampling employed for qualitative data?

***Response: We now clarify how facilities were selected for inclusion in the methods section. We also clarify that convenience sampling was employed for recruitment of FGD participants who were invited from antenatal care services.***

- **Qualitative Data Collection and Analysis**: For Focus Group Discussions (FGDs), please clarify the data analysis process. Specify if coding was conducted and whether a thematic analysis was used.

***Response: The qualitative data analysis methods have been more thoroughly described. As described in the text, deductive thematic analysis was used, without coding.***

\#### Results
Clearly separate the findings into quantitative and qualitative sections. Explain how the results from the two study designs intersect—whether this occurs during data analysis or interpretation.

***Response: The results from qualitative and quantitative are now presented in separate sections. The quantitative and qualitative data converge at the interpretation stage.***

The qualitative findings are inadequately reported. Please adhere to
Consolidated criteria for reporting qualitative research (COREQ)
guidelines and ensure the following:
- State whether saturation of information was reached.
- Provide a code tree with clear themes, sub-themes, and representative quotes for each theme.
- Ensure the voices of both majority and minority participants, including both negative and positive perspectives, are represented.
Please also submit the COREQ checklist as a supplemental file in your next revision

***Response: Each of these items have now been described and/or reported in the manuscript. The COREQ checklist is completed and attached.***

\#### Discussion
Begin this section by summarizing the key findings and the main messages readers should retain.

***Response: The discussion now begins with a summary of the key findings/main messages.***

The current discussion is overly simplistic. Please elaborate on each key finding, including its implications, and compare these results with studies from other countries to provide a global perspective.

***Response: We now further contextualize our findings in light of prior literature in the in the region.***

Additionally, a limitations section is missing and should be included.

***Response: A limitations section has been added.***

Please also add a recommendations section, rooted in key findings, that addresses potential policy implications.

\#### Conclusion
Revise the conclusion to remind readers of the key findings and recommendations in a concise manner.

***Response:* The conclusions section has been revised to reiterate the key findings and to make recommendations rooted in findings.**

Thank you for considering these suggestions to enhance the manuscript's clarity and impact.

**Reviewer #2:** The research focuses on a very important topic, but unfortunately the methodology (sampling, inclusion, & exclusion criteria and narration of mix methodology) as well as the results require narration with a lot more clarity. Moreover, consistency and flow of the narration needs improvement. Therefore would advise a major review.

**Response: We appreciate this comment and hope that the revisions and edits noted above and within the manuscript address these issues.**

6. PLOS authors have the option to publish the peer review history of their article ([what does this mean?](https://nam11.safelinks.protection.outlook.com/?url=https%3A%2F%2Ftrack.editorialmanager.com%2FCL0%2Fhttps%3A%252F%252Fjournals.plos.org%252Fplosone%252Fs%252Feditorial-and-peer-review-process%2523loc-peer-review-history%2F1%2F010f01934be440e1-7c66cd29-772a-4590-9e5b-f081716821a5-000000%2F4cbxPxtR8CaOsyDwBR6K72bopKvhVAtoGX6hmjXbieg%3D186&data=05%7C02%7Ckmwall%40emory.edu%7C6b139e4f7a694861d73208dd09ba7c6b%7Ce004fb9cb0a4424fbcd0322606d5df38%7C0%7C0%7C638677419212816457%7CUnknown%7CTWFpbGZsb3d8eyJFbXB0eU1hcGkiOnRydWUsIlYiOiIwLjAuMDAwMCIsIlAiOiJXaW4zMiIsIkFOIjoiTWFpbCIsIldUIjoyfQ%3D%3D%7C0%7C%7C%7C&sdata=GjmGYHzIaTK1hZkWFYJXciBiuOvM4HqU%2B33kuoNoRyM%3D&reserved=0)). If published, this will include your full peer review and any attached files.


If you choose “no”, your identity will remain anonymous but your review may still be made public.


**Do you want your identity to be public for this peer review?** For information about this choice, including consent withdrawal, please see our [Privacy Policy](https://nam11.safelinks.protection.outlook.com/?url=https%3A%2F%2Ftrack.editorialmanager.com%2FCL0%2Fhttps%3A%252F%252Fwww.plos.org%252Fprivacy-policy%2F1%2F010f01934be440e1-7c66cd29-772a-4590-9e5b-f081716821a5-000000%2FxpLN1tJyrJI9ZiIjv3fu-GXxEAQwi2bzX-CpR9A6gbo%3D186&data=05%7C02%7Ckmwall%40emory.edu%7C6b139e4f7a694861d73208dd09ba7c6b%7Ce004fb9cb0a4424fbcd0322606d5df38%7C0%7C0%7C638677419212829575%7CUnknown%7CTWFpbGZsb3d8eyJFbXB0eU1hcGkiOnRydWUsIlYiOiIwLjAuMDAwMCIsIlAiOiJXaW4zMiIsIkFOIjoiTWFpbCIsIldUIjoyfQ%3D%3D%7C0%7C%7C%7C&sdata=8w9MLSrhgrqG31tISDoFCrK2nlOid2F%2FUH%2FfCf46zxE%3D&reserved=0).

Reviewer #1: No

Reviewer #2: No

[NOTE: If reviewer comments were submitted as an attachment file, they will be attached to this email and accessible via the submission site. Please log into your account, locate the manuscript record, and check for the action link "View Attachments". If this link does not appear, there are no attachment files.]

While revising your submission, please upload your figure files to the Preflight Analysis and Conversion Engine (PACE) digital diagnostic tool, [https://pacev2.apexcovantage.com/](https://nam11.safelinks.protection.outlook.com/?url=https%3A%2F%2Ftrack.editorialmanager.com%2FCL0%2Fhttps%3A%252F%252Fpacev2.apexcovantage.com%252F%2F1%2F010f01934be440e1-7c66cd29-772a-4590-9e5b-f081716821a5-000000%2F9zsWrWcma0Tha_00b0TINCz2vDlC8QJVBx5G1PpTy5g%3D186&data=05%7C02%7Ckmwall%40emory.edu%7C6b139e4f7a694861d73208dd09ba7c6b%7Ce004fb9cb0a4424fbcd0322606d5df38%7C0%7C0%7C638677419212842103%7CUnknown%7CTWFpbGZsb3d8eyJFbXB0eU1hcGkiOnRydWUsIlYiOiIwLjAuMDAwMCIsIlAiOiJXaW4zMiIsIkFOIjoiTWFpbCIsIldUIjoyfQ%3D%3D%7C0%7C%7C%7C&sdata=SvYEYRxw1JLgIcZ2lsfm5aOWZ90FTsovlBZ11tRJUMs%3D&reserved=0). PACE helps ensure that figures meet PLOS requirements. To use PACE, you must first register as a user. Registration is free. Then, login and navigate to the UPLOAD tab, where you will find detailed instructions on how to use the tool. If you encounter any issues or have any questions when using PACE, please email PLOS at [figures@plos.org](mailto:figures@plos.org). Please note that Supporting Information files do not need this step.
