# Supplementary material for: Implementing postpartum family planning services in rural Rwanda: A mixed-methods study
Source: PLoS One. 2025 Dec 30;20(12):e0338627. doi: 10.1371/journal.pone.0338627 (PMC12752963; doi:10.1371/journal.pone.0338627)
Supplement: Attachment — Submitted filename: RuralPPFP_ReviewerResponse.docx [file pone.0338627.s007.docx]

PONE-D-24-28650R1

Implementing postpartum family planning services in rural Rwanda: a mixed-methods study

PLOS ONE

Dear Dr. Wall,

Thank you for submitting your manuscript to PLOS ONE. After careful consideration, we feel that it has merit but does not fully meet PLOS ONE’s publication criteria as it currently stands. Therefore, we invite you to submit a revised version of the manuscript that addresses the points raised during the review process.

Based on the outcome of peer-review, the manuscript requires to undergo major revisions before the journal can further considers it.

Please submit your revised manuscript by Oct 10 2025 11:59PM. If you will need more time than this to complete your revisions, please reply to this message or contact the journal office at [plosone@plos.org](mailto:plosone@plos.org). When you're ready to submit your revision, log on to https://www.editorialmanager.com/pone/ and select the 'Submissions Needing Revision' folder to locate your manuscript file.

Please include the following items when submitting your revised manuscript:

- A rebuttal letter that responds to each point raised by the academic editor and reviewer(s). You should upload this letter as a separate file labeled 'Response to Reviewers'.
- A marked-up copy of your manuscript that highlights changes made to the original version. You should upload this as a separate file labeled 'Revised Manuscript with Track Changes'.
- An unmarked version of your revised paper without tracked changes. You should upload this as a separate file labeled 'Manuscript'.

If you would like to make changes to your financial disclosure, please include your updated statement in your cover letter. Guidelines for resubmitting your figure files are available below the reviewer comments at the end of this letter.

If applicable, we recommend that you deposit your laboratory protocols in protocols.io to enhance the reproducibility of your results. Protocols.io assigns your protocol its own identifier (DOI) so that it can be cited independently in the future. For instructions see: [https://journals.plos.org/plosone/s/submission-guidelines#loc-laboratory-protocols](https://nam11.safelinks.protection.outlook.com/?url=https%3A%2F%2Ftrack.editorialmanager.com%2FCL0%2Fhttps%3A%252F%252Fjournals.plos.org%252Fplosone%252Fs%252Fsubmission-guidelines%2523loc-laboratory-protocols%2F1%2F010f0198e63a0634-5bf5854d-d738-4901-847d-b29d072ff178-000000%2FfbGH61iUk2WSUxzk5apn98Sr_QJWD5FpwtYlgqKGom8%3D225&data=05%7C02%7Ckmwall%40emory.edu%7Cc2744db26cce42a1da5d08dde49732dc%7Ce004fb9cb0a4424fbcd0322606d5df38%7C0%7C0%7C638918060390961781%7CUnknown%7CTWFpbGZsb3d8eyJFbXB0eU1hcGkiOnRydWUsIlYiOiIwLjAuMDAwMCIsIlAiOiJXaW4zMiIsIkFOIjoiTWFpbCIsIldUIjoyfQ%3D%3D%7C0%7C%7C%7C&sdata=2h9PwPVCRDzpH1TpkMJaMy80EG7%2BVS4tBWhKzKYMSmU%3D&reserved=0). Additionally, PLOS ONE offers an option for publishing peer-reviewed Lab Protocol articles, which describe protocols hosted on protocols.io. Read more information on sharing protocols at [https://plos.org/protocols?utm_medium=editorial-email&utm_source=authorletters&utm_campaign=protocols](https://nam11.safelinks.protection.outlook.com/?url=https%3A%2F%2Ftrack.editorialmanager.com%2FCL0%2Fhttps%3A%252F%252Fplos.org%252Fprotocols%253Futm_medium%3Deditorial-email%2526utm_source%3Dauthorletters%2526utm_campaign%3Dprotocols%2F1%2F010f0198e63a0634-5bf5854d-d738-4901-847d-b29d072ff178-000000%2F-5rOj12PNfV3gMku1yjK9EV4aW34dBiSIBSo4sorKxc%3D225&data=05%7C02%7Ckmwall%40emory.edu%7Cc2744db26cce42a1da5d08dde49732dc%7Ce004fb9cb0a4424fbcd0322606d5df38%7C0%7C0%7C638918060390982800%7CUnknown%7CTWFpbGZsb3d8eyJFbXB0eU1hcGkiOnRydWUsIlYiOiIwLjAuMDAwMCIsIlAiOiJXaW4zMiIsIkFOIjoiTWFpbCIsIldUIjoyfQ%3D%3D%7C0%7C%7C%7C&sdata=jjoltEp%2BAhmTk%2FvVDLwNQY%2BZwvMI8kjbi6NSjLoAnP8%3D&reserved=0).

We look forward to receiving your revised manuscript.

Kind regards,

Syed Khurram Azmat, PhD, MPH, MD
Academic Editor
PLOS ONE

Journal Requirements:

If the reviewer comments include a recommendation to cite specific previously published works, please review and evaluate these publications to determine whether they are relevant and should be cited. There is no requirement to cite these works unless the editor has indicated otherwise. 


[Note: HTML markup is below. Please do not edit.]

Reviewers' comments:

Reviewer's Responses to Questions

**Comments to the Author**

1. If the authors have adequately addressed your comments raised in a previous round of review and you feel that this manuscript is now acceptable for publication, you may indicate that here to bypass the “Comments to the Author” section, enter your conflict of interest statement in the “Confidential to Editor” section, and submit your "Accept" recommendation.

Reviewer #1: (No Response)

Reviewer #3: All comments have been addressed

2. Is the manuscript technically sound, and do the data support the conclusions?

The manuscript must describe a technically sound piece of scientific research with data that supports the conclusions. Experiments must have been conducted rigorously, with appropriate controls, replication, and sample sizes. The conclusions must be drawn appropriately based on the data presented.

Reviewer #1: Partly

Reviewer #3: Partly

3. Has the statistical analysis been performed appropriately and rigorously?

Reviewer #1: Yes

Reviewer #3: No

4. Have the authors made all data underlying the findings in their manuscript fully available?

The [PLOS Data policy](https://nam11.safelinks.protection.outlook.com/?url=http%3A%2F%2Ftrack.editorialmanager.com%2FCL0%2Fhttp%3A%252F%252Fwww.plosone.org%252Fstatic%252Fpolicies.action%2523sharing%2F1%2F010f0198e63a0634-5bf5854d-d738-4901-847d-b29d072ff178-000000%2FYfVbktPwE5QaOKIsV_4ry40gpD7-mHaMXxNv9XV5w_E%3D225&data=05%7C02%7Ckmwall%40emory.edu%7Cc2744db26cce42a1da5d08dde49732dc%7Ce004fb9cb0a4424fbcd0322606d5df38%7C0%7C0%7C638918060390999014%7CUnknown%7CTWFpbGZsb3d8eyJFbXB0eU1hcGkiOnRydWUsIlYiOiIwLjAuMDAwMCIsIlAiOiJXaW4zMiIsIkFOIjoiTWFpbCIsIldUIjoyfQ%3D%3D%7C0%7C%7C%7C&sdata=XjjwBWje1ZaddtXExrK5d3OZNgk%2BdkK0wnV9jBBF8kw%3D&reserved=0) requires authors to make all data underlying the findings described in their manuscript fully available without restriction, with rare exception (please refer to the Data Availability Statement in the manuscript PDF file). The data should be provided as part of the manuscript or its supporting information, or deposited to a public repository. For example, in addition to summary statistics, the data points behind means, medians and variance measures should be available. If there are restrictions on publicly sharing data—e.g. participant privacy or use of data from a third party—those must be specified.

Reviewer #1: Yes

Reviewer #3: Yes

5. Is the manuscript presented in an intelligible fashion and written in standard English?

PLOS ONE does not copyedit accepted manuscripts, so the language in submitted articles must be clear, correct, and unambiguous. Any typographical or grammatical errors should be corrected at revision, so please note any specific errors here.

Reviewer #1: Yes

Reviewer #3: Yes

6. Review Comments to the Author

Please use the space provided to explain your answers to the questions above. You may also include additional comments for the author, including concerns about dual publication, research ethics, or publication ethics. (Please upload your review as an attachment if it exceeds 20,000 characters)

Reviewer #1: Thank you for the opportunity to review the revised manuscript. The manuscript has improved remarkably, and the authors have addressed most of the previous comments. However, the research implications remain unaddressed, and the recommendation section has been entirely omitted. This is a significant oversight. Additionally, the manuscript reflects a lack of experience and understanding in reporting qualitative research, particularly in the presentation of qualitative findings, which require major revision.
Below are other specific comments for your consideration:

Abstract
Methods – Avoid presenting numerical data in the abstract for qualitative research. Instead, summarize the key themes that emerged from the study and highlight the main takeaway message. Simply stating, “The majority of the 27 couples recruited for FGDs (93% of women, 74% of men) had received family planning educational counseling though less than half (41%) of couples reported learning about the PPIUD” does not adequately reflect the richness and depth of the qualitative findings. This approach oversimplifies the data and misses the nuanced perspectives shared by participants. Emphasis should be placed on the insights gained about participants’ experiences, perceptions, and barriers related to family planning and PPIUD counseling.

**Response**: ***We have reviewed the results section of the abstract, and the qualitative findings are now presented as themes highlighting the main takeaway messages of our study.***

Introduction
Since the introduction mentions both the cost of contraceptives and Rwanda’s promotion of postpartum family planning (PPFP) and long-acting reversible contraception (LARC), please include a brief context on how these services are accessed in practice. Are they freely available through the public health system in Rwanda? Adding one or two sentences—ideally with a valid reference—about the availability and affordability of PPFP and LARC would provide a more complete picture. This addition could be placed in either the second or third paragraph of the introduction without disrupting the narrative flow.

**Response: *We have briefly described how affordable and available family planning (FP) services are in Rwanda in the 3^rd^ paragraph. Briefly, FP services are provided in both public and private settings and are covered by public health insurance which provides essentially universal healthcare. Refills of short term methods are provided free of charge in the community by community health workers.***

Implication of the study
While the authors responded that they have added the implications of the study, I do not see these changes reflected in the current version of the introduction. The section still lacks a clear articulation of the research gap. Although structural barriers are mentioned, it remains unclear whether similar studies on postpartum family planning (PPFP) or PPIUD have been conducted in Rwanda before. If such studies exist, what gap does this research address? What new insights does it contribute to the existing evidence base? If no similar studies exist, why is this study needed, and how can it inform policy, practice, or future research? I recommend elaborating on these points to clearly establish the significance and contribution of this study. Please add this to the last paragraph of the introduction section, just before the aims of the study.

**Response: *We have added a paragraph in the introduction highlighting the impact of our study. While the few FP studies conducted in rural Rwanda have confirmed the low uptake of PPFP, there has not been an assessment of rural facility/staff readiness in offering PPFP services along with both women and their male partners perceptions of PPFP needs and services.***

Methods
Line 141 – The sampling method used for the FGDs appears to be purposive rather than convenience sampling. There are important nuances between these two approaches: purposive sampling involves deliberately selecting participants based on specific characteristics relevant to the research question, while convenience sampling relies on accessibility and availability. Please review the definitions of both methods and revise the manuscript accordingly if purposive sampling was indeed used.

**Response: *We confirm that our sampling method used for the FGDs was convenience sampling.*** ***As explained from Line 145-157, the study team selected high volume, accessible and non-Catholic facilities in the two rural districts, and all adult pregnant women coming with their partner were recruited for FGDs from ANC services. No additional criteria were used to purposively select participants for focus groups.***

Line 173 – correct the word “propose” to “purpose”.

**Response: *This type has been corrected.***

Line 197 – In qualitative research, while we may have a general idea of the approximate sample size needed, it is not rigidly predetermined as in quantitative studies. Since your data collection continued until the point of saturation, it would be more appropriate to simply state that. Please revise the sentence “The sample size was pre-specified to be sufficient to reach saturation based on prior guidelines for saturation”—as this is technically not accurate for qualitative research. A more appropriate phrasing could be: “Data collection continued until thematic saturation was reached, in line with qualitative research principles.”

**Response: *Line 209 was updated accordingly and we removed the previous statement as suggested.***

Qualiative findingss
The reporting of qualitative findings still requires improvement.
Please avoid presenting percentages when describing responses. In qualitative research, we focus on the depth and meaning of participants’ experiences rather than quantifying how many said what. Using terms like “majority” or providing percentages undermines the interpretive nature of qualitative data.

Moreover, the code tree or thematic structure is missing, which is an important component recommended by the COREQ (Consolidated Criteria for Reporting Qualitative Research) guidelines. Please include a table outlining the main themes and their corresponding subthemes or categories. Even if the themes were predefined, the findings should still be clearly structured under each one to improve readability and coherence. At present, the lack of an organized thematic framework makes the results difficult to follow.
Since you’ve indicated that the Theory of Planned Behavior guided the study, consider aligning the themes with its core constructs. This would not only enhance theoretical consistency but also strengthen the interpretive value of your findings.

The predefined themes outlined in the manuscript provide limited insight into the how and why behind participants’ experiences and thus offer very little depth. To fully capture the richness of the qualitative data, the analysis should move beyond superficial descriptions and explore underlying meanings, processes, and contextual factors that shape participants’ perspectives.
The reporting of qualitative findings requires major revision to adequately reflect the depth, complexity, and richness of the data.

**Response: *The code tree is now added, and the qualitative results have been rearranged based on themes emerging from the data.***

Discussion-limitations
Lines 437–439 – This statement does not represent a true limitation. The note that illustrative quotes cannot be linked to participants’ age, number of pregnancies, children, or previous use of PPFP is unnecessary. In qualitative research, it is common practice to provide contextual information such as participant gender, age group, or parity when presenting quotes, without necessarily linking every response to detailed demographic data. I suggest revising this section to reflect that quotes can still be meaningfully contextualized by key participant characteristics like gender and parity, which would enrich the findings rather than constitute a limitation.

Please revise the limitations section. The points currently listed do not represent valid limitations of the study. Instead, consider discussing methodological constraints such as reliance on existing quantitative databases with limited variables, potential recall bias, or challenges related to the study timeline and data collection period. Reflecting on these practical and contextual limitations will provide a more meaningful and balanced assessment of the study’s scope and potential weaknesses.

**Response: *We do believe that by recruiting participants right after attending ANC visits where FP counseling messages are discussed that the study team has mitigated recall bias. We do however recognize that social desirability bias would be possible in this study.*** ***The limitations section has been revised accordingly.***

Recommendations
The manuscript did not address my earlier comment regarding the recommendations section, which is currently missing. Please include a dedicated recommendations section that clearly highlights how the findings can inform policymakers, practitioners working in postpartum family planning (PPFP), and future research in this area. Ensure that these recommendations are directly rooted in and supported by the study’s findings to enhance their relevance and practical utility.

**Response: *The recommendations section was revised to include recommendations for stakeholders and practitioners as well as future investigations needed to sustain PPFP uptake overtime.***

Reviewer #3: This paper is interesting, but has a fair bit of revision necessary in order to be publishable.

As a minor point, there are too many writing and grammatical errors, particularly numerical inconsistencies (six instead of 6).

**Response: *The manuscript was proofread, and grammatical errors were addressed.***

More substantively, it would be helpful to know more about these two regions of Rwanda. How to they compare to others in terms of population density, prior FP use, ethnic diversity (e.g. refugee populations from surrounding nations). Outside of Kigali much of the nation is rural, but there is still demographic diversity so more information on why these two regions are selected (beyond convenience) would be helpful to contextualize the results.

**Response**: ***A short paragraph on the selection of the two rural district was added****.*

The quantitative analysis is somewhat limited in terms of comparisons. If some clinics/hospitals/health posts have limited capabilities for PPFP, is that consistent with other regions? Of course 100% is ideal, but what is realistic, and how far are these numbers from the realistic goal?

Qualitative focus groups should include more information as to how data was collected. Were husbands and wives in the same room at the same time? Could this influence responses? It is a real strength of this paper to include male voices in family planning conversations, but much more depth and details is needed to explain the process through which this data was collected, and the potential sources of bias and error in a highly patriarchal culture.

**Response**: ***We have added to the methods section a description about how the couple FGDs were conducted to help the reader better understand the data collection process.***

Most importantly, what information are we gleaning from this study beyond the idea that more resources, training, and information would improve PPFP outcomes. What can we learn from this that would help Rwandan communities specifically and public health scholars more generally?

**Response: *Our study impact and contributions is highlighted in the introduction section.***

7. PLOS authors have the option to publish the peer review history of their article ([what does this mean?](https://nam11.safelinks.protection.outlook.com/?url=https%3A%2F%2Ftrack.editorialmanager.com%2FCL0%2Fhttps%3A%252F%252Fjournals.plos.org%252Fplosone%252Fs%252Feditorial-and-peer-review-process%2523loc-peer-review-history%2F1%2F010f0198e63a0634-5bf5854d-d738-4901-847d-b29d072ff178-000000%2FT6CUrOni0f44_0QyR93sSBxN14UCM1SlwOr0C7OjlTY%3D225&data=05%7C02%7Ckmwall%40emory.edu%7Cc2744db26cce42a1da5d08dde49732dc%7Ce004fb9cb0a4424fbcd0322606d5df38%7C0%7C0%7C638918060391016668%7CUnknown%7CTWFpbGZsb3d8eyJFbXB0eU1hcGkiOnRydWUsIlYiOiIwLjAuMDAwMCIsIlAiOiJXaW4zMiIsIkFOIjoiTWFpbCIsIldUIjoyfQ%3D%3D%7C0%7C%7C%7C&sdata=ay%2FPkv85gOuVXEqcnT56ObIUTWF2vN%2BsFROmn1EfsDo%3D&reserved=0)). If published, this will include your full peer review and any attached files.

If you choose “no”, your identity will remain anonymous but your review may still be made public.

**Do you want your identity to be public for this peer review?** For information about this choice, including consent withdrawal, please see our [Privacy Policy](https://nam11.safelinks.protection.outlook.com/?url=https%3A%2F%2Ftrack.editorialmanager.com%2FCL0%2Fhttps%3A%252F%252Fwww.plos.org%252Fprivacy-policy%2F1%2F010f0198e63a0634-5bf5854d-d738-4901-847d-b29d072ff178-000000%2FlL89QIbJ7B0c1y6edpnsBwrYZtXH2K5PoOay-rDq6aE%3D225&data=05%7C02%7Ckmwall%40emory.edu%7Cc2744db26cce42a1da5d08dde49732dc%7Ce004fb9cb0a4424fbcd0322606d5df38%7C0%7C0%7C638918060391033178%7CUnknown%7CTWFpbGZsb3d8eyJFbXB0eU1hcGkiOnRydWUsIlYiOiIwLjAuMDAwMCIsIlAiOiJXaW4zMiIsIkFOIjoiTWFpbCIsIldUIjoyfQ%3D%3D%7C0%7C%7C%7C&sdata=YTSVe9vh1BytAs4fd9kO1rLsZEp6HAV9scmfN76Ilzc%3D&reserved=0).

Reviewer #1: No

Reviewer #3: No

[NOTE: If reviewer comments were submitted as an attachment file, they will be attached to this email and accessible via the submission site. Please log into your account, locate the manuscript record, and check for the action link "View Attachments". If this link does not appear, there are no attachment files.]

While revising your submission, please upload your figure files to the Preflight Analysis and Conversion Engine (PACE) digital diagnostic tool, [https://pacev2.apexcovantage.com/](https://nam11.safelinks.protection.outlook.com/?url=https%3A%2F%2Ftrack.editorialmanager.com%2FCL0%2Fhttps%3A%252F%252Fpacev2.apexcovantage.com%252F%2F1%2F010f0198e63a0634-5bf5854d-d738-4901-847d-b29d072ff178-000000%2FWGv-iik0kOC99BvDCkmPevg3FGbA7E6GMlSk1zoE-7E%3D225&data=05%7C02%7Ckmwall%40emory.edu%7Cc2744db26cce42a1da5d08dde49732dc%7Ce004fb9cb0a4424fbcd0322606d5df38%7C0%7C0%7C638918060391049477%7CUnknown%7CTWFpbGZsb3d8eyJFbXB0eU1hcGkiOnRydWUsIlYiOiIwLjAuMDAwMCIsIlAiOiJXaW4zMiIsIkFOIjoiTWFpbCIsIldUIjoyfQ%3D%3D%7C0%7C%7C%7C&sdata=0oHOZ0SPy53kI8d8M6vFGv85P1ErxUeyIH1AzNv94OQ%3D&reserved=0). PACE helps ensure that figures meet PLOS requirements. To use PACE, you must first register as a user. Registration is free. Then, login and navigate to the UPLOAD tab, where you will find detailed instructions on how to use the tool. If you encounter any issues or have any questions when using PACE, please email PLOS at [figures@plos.org](mailto:figures@plos.org). Please note that Supporting Information files do not need this step.
